# Delivery of Apoplastic Extracellular Vesicles Encapsulating Green-Synthesized Silver Nanoparticles to Treat Citrus Canker

**DOI:** 10.3390/nano13081306

**Published:** 2023-04-07

**Authors:** Isha Gaurav, Abhimanyu Thakur, Gaurav Kumar, Qin Long, Kui Zhang, Rakesh Kumar Sidu, Sudha Thakur, Rajesh Kumar Sarkar, Anoop Kumar, Ashok Iyaswamy, Zhijun Yang

**Affiliations:** 1 School of Chinese Medicine, Hong Kong Baptist University, Hong Kong SAR 999077, China; 2 Ben May Department for Cancer Research, Pritzker School of Molecular Engineering, University of Chicago, Chicago, IL 60637, USA; 3 Clinical Research Division, Department of Biosciences, School of Basic and Applied Sciences, Galgotias University, Greater Noida 203201, Uttar Pradesh, India; 4 Citrus Research Institute, Southwest University, Chinese Academy of Agricultural Sciences, National Citrus Engineering Research Center, Chongqing 400712, China; 5 School of Biomedical Engineering, Indian Institute of Technology, Banaras Hindu University, Varanasi 221005, Uttar Pradesh, India; 6 National Institute for Locomotor Disabilities (Divyangjan), Kolkata 700090, India; 7 Department of Medicine, Division of Biological Sciences, University of Chicago, Chicago, IL 60637, USA; 8 Department of Pharmacology, Delhi Pharmaceutical Sciences and Research University (DPSRU), New Delhi 110017, India; 9 Mr. & Mrs. Ko Chi-Ming Centre for Parkinson’s Disease Research, School of Chinese Medicine, Hong Kong Baptist University, Hong Kong SAR 999077, China; 10 Department of Biochemistry, Karpagam Academy of Higher Education, Coimbatore 641021, India; 11 Changshu Research Institute, Hong Kong Baptist University, Changshu Economic and Technological Development (CETD) Zone, Changshu 215500, China

**Keywords:** citrus canker, drug delivery, green synthesis, silver nanoparticles, *Phyllanthus niruri*, antimicrobial activity, *Xanthomonas axonopodis*, nirurinetin

## Abstract

The citrus canker pathogen *Xanthomonas axonopodis* has caused severe damage to citrus crops worldwide, resulting in significant economic losses for the citrus industry. To address this, a green synthesis method was used to develop silver nanoparticles with the leaf extract of *Phyllanthus niruri* (GS-AgNP-LEPN). This method replaces the need for toxic reagents, as the LEPN acts as a reducing and capping agent. To further enhance their effectiveness, the GS-AgNP-LEPN were encapsulated in extracellular vesicles (EVs), nanovesicles with a diameter of approximately 30–1000 nm naturally released from different sources, including plant and mammalian cells, and found in the apoplastic fluid (APF) of leaves. When compared to a regular antibiotic (ampicillin), the delivery of APF-EV-GS-AgNP-LEPN and GS-AgNP-LEPN to *X. axonopodis* pv. was shown to have more significant antimicrobial activity. Our analysis showed the presence of phyllanthin and nirurinetin in the LEPN and found evidence that both could be responsible for antimicrobial activity against *X. axonopodis* pv. Ferredoxin-NADP^+^ reductase (FAD-FNR) and the effector protein XopAI play a crucial role in the survival and virulence of *X. axonopodis* pv. Our molecular docking studies showed that nirurinetin could bind to FAD-FNR and XopAI with high binding energies (−10.32 kcal/mol and −6.13 kcal/mol, respectively) as compared to phyllanthin (−6.42 kcal/mol and −2.93 kcal/mol, respectively), which was also supported by the western blot experiment. We conclude that (a) the hybrid of APF-EV and GS-NP could be an effective treatment for citrus canker, and (b) it works via the nirurinetin-dependent inhibition of FAD-FNR and XopAI in *X. axonopodis* pv.

## 1. Introduction

Citrus canker is a serious infectious disease occurring in citrus species due to *Xanthomonas axonopodis* pv. It causes dark spots and lesions on the leaves, stem, and fruit of, e.g., limes, oranges, and grapefruit [1,2]. *X. axonopodis* pv. also causes the bacterial blight of cassava [3]. Severe infection by *X. axonopodis* pv. can lead to defoliation, premature fruit drop, blemished fruit, twig die-back, and weakening of the tree, resulting in a great economic loss [4]. There are various functional proteins found in *X. axonopodis* pv., which play a crucial role in the pathogenic progression of citrus canker (Supplementary Table S1), and these are potential targets for its treatment [5,6,7,8,9,10,11,12,13]. Two current treatment options are phage treatment [14] and biocontrol by using an endophytic *Bacillus thuringiensis* [15]. Combinatorial management practices are also utilized. These include the careful cultivation of disease-free nursery plants, the removal of infected twigs, the application of copper-based bactericidal sprays, and the development of canker-resistant varieties. A combination of copper oxychloride (0.3%), streptocycline (100 ppm), and neem cake suspension has been found to be effective when sprayed on infected twigs [15]. The antibiotic streptomycin has been used to control various plant diseases including bacterial blight and spot [15,16]; however, the citrus canker pathogen has become resistant to streptomycin owing to its excessive and prolonged application [17,18]. Despite the many treatments tried and applied, none are consistently effective. Therefore, the exploration of new strategies is needed [19]. 

**Scheme 1:**
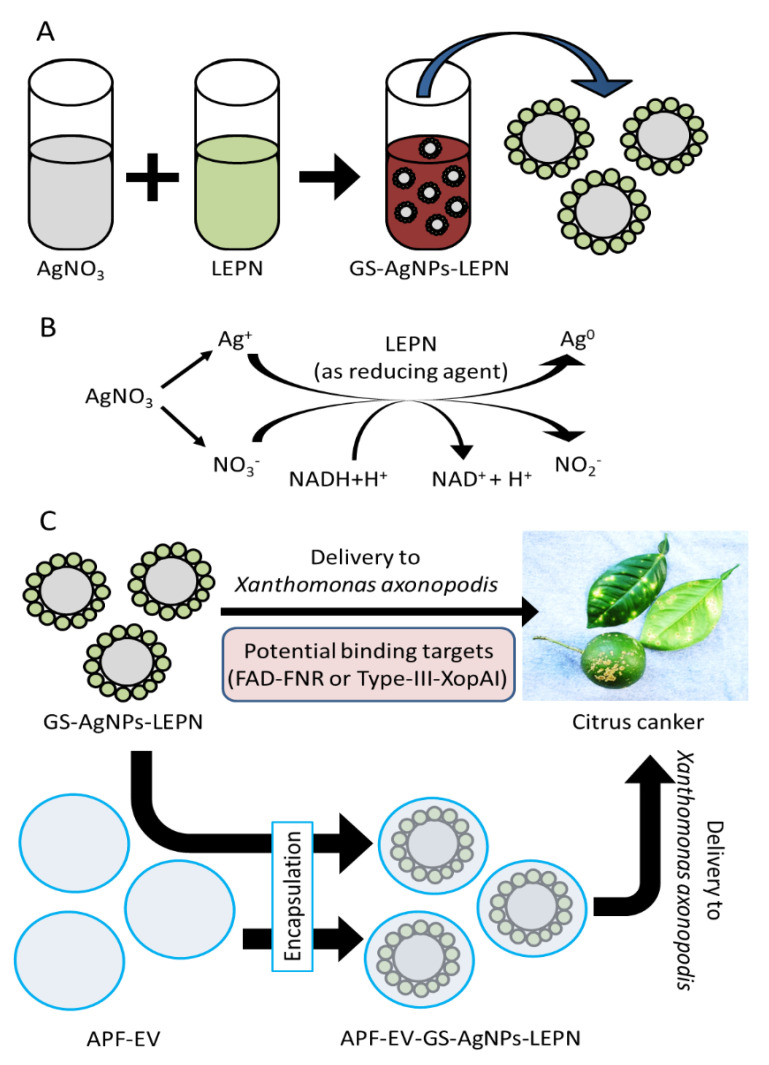
Production and delivery of GS-AgNP-LEPN to treat citrus canker. (**A**) Leaf extract of *Phyllanthus niruri* (LEPN) is mixed with AgNO_3_ solution. (**B**) Reduction of Ag+ in the presence of LEPN, acting as a reducing agent to form GS-AgNP-LEPN. (**C**) GS-AgNP-LEPN and APF-EV-GS- AgNP-LEPN are delivered to *X. axonopodis* pv. infecting citrus leaves and fruit. In-set: Photograph of symptoms of citrus canker caused by the bacterium *X. axonopodis* pv. On the leaves and fruits of citrus plants growing in the Federated States of Micronesia (Photo: Scot C. Nelson via Flickr).

Recently, the application of nanoparticles (NP) has attracted attention as a novel means to control various plant diseases including citrus canker. NP have various advantages; for example, they are highly reactive, can be readily formulated for specific purposes, and have anti-microbial properties [20,21]. Several metallic NP (MNP) have been developed and demonstrated to have antibacterial properties, as listed in Supplementary Table S2 [22,23,24,25,26,27,28]. Compared to conventional pesticides, MNP have less toxicity and a longer shelf-life. Further, they can be used to deliver cargo that is otherwise poorly soluble in water [29]. However, the synthesis of MNP involves the use of chemical-reducing agents needed to convert metal ions to MNP; in other words, the synthesis involves hazardous and toxic chemicals [30,31]. Therefore, the green synthesis of NP using various plant parts has become a new direction of research. If plant parts including the leaf, stem, and roots are used as reducing and capping agents, the process becomes environmentally friendly and sustainable, eliminating toxic residues [32] and reducing waste [33]. Green synthesis can provide stable NP via a simple, cost-effective, and reproducible process that can be readily scaled up [32]. Therefore, in the present research, we have developed green-synthesized (GS)-silver NP with the leaf extract of *Phyllanthus niruri* (GS-AgNP-LEPN) (Scheme 1A,B). As far as we know, nobody has investigated the antimicrobial activity of GS-AgNP-LEPN for treating citrus canker.

Biomimetic NP retain distinctive characteristics of natural biomaterials, including cell membranes. Green-synthesized NP have attracted dramatic interest as an efficient biocompatible drug delivery system [34,35,36]. The membranes of different types of cells (e.g., red blood cells, cancer cells, platelets) can be coated with NP, and NP-coated cells have shown excellent biocompatibility [37]. Extracellular vesicles (EVs) are intraluminal nanovesicles bearing various cargoes including nucleic acids and proteins released from various cell types and different parts of plants [34,35]. Plant-derived EV have appeal as nanocarriers due to advantages that include higher biocompatibility, low toxicity, low immunogenicity, and innocuity. Autologous EV possess an effective homing capacity [38]. EV have been found in apoplastic fluids (APF; usually extracted from plant leaves) [39]; however their potential as nano delivery agents has not been investigated. In this study, for the first time, we isolated autologous EV from the APF of the leaf of *Phyllanthus niruri* and encapsulated GS-AgNP-LEPN to produce EV-GS-AgNP-LEPN, followed by an examination of antibacterial efficacy.

*P. niruri* (common names: Bhumi amla, Nela usari, Pitirishi, or Bukhari in the traditional Indian medicine system; stonebreaker; gale of the wind; seed-under-leaf in English, Zhu zi cao in Chinese, Chanca Piedra in Spanish, Quebra Pedra in Brazil, Quinine creole in French, Kidachi komi kansou in Japanese, Quebra-pedra in Portuguese; synonym, *P. amarus*; *Euphorbiaceae*) is widely used across the world as a medicine [40,41]. Various species of *Phyllanthus* including *P. niruri* have been widely studied for their potential antimicrobial properties against different phytopathogens (Supplementary Table S3) [42,43,44,45,46,47,48,49,50,51]. However, the mechanism of this activity has not been studied. Similarly, while some of the active constituents of *P. niruri* (e.g., phyllanthin) have been studied for their antibacterial activity, nirurinetin has not been studied so far. 

The ferredoxin-NADP^+^ reductase (FNR) of *X. axonopodis* pv. citri plays a crucial role in maintaining the host–pathogen infection [12,52,53]. In addition, the effector protein XopAI has been found to be responsible for the survival and virulence of *X. axonopodis* pv. [54]. Therefore, these two could be important antibacterial targets. 

In this study, we developed a novel APF-EV encapsulating GS-AgNP-LEPN for the first time and evaluated its antimicrobial activity against *X. axonopodis* pv., a microorganism causing citrus canker. We found significant antimicrobial activity of APF-EV encapsulating GS-AgNP-LEPN against *X. axonopodis* pv. and hypothesized that nirurinetin could act on FNR and the effector protein XopAI for the antibacterial activity. To further elucidate the mechanism of action, we outlined the rationale and procedure of this study in Scheme 1C. This phyto nano-drug delivery strategy may be extendable to combatting other plant or human microorganisms.

## 2. Materials and Methods

### 2.1. Chemicals and Consumables

AgNO_3_, Standard Phyllanthin, Potassium Bromide (KBr) (98% purity HPLC grade from Natural Remedies Pvt. Ltd., Bengaluru, India), acetonitrile (Finar Chemicals Ltd., Ahmedabad, India), methanol (Finar Chemicals Ltd., Gujarat, India), carbon tape, a mercury lamp, and a carbon-coated copper grid (local vendor in India) were used. 

### 2.2. Collection and Pre-Processing of the Plant Sample

A fresh and healthy plant of *P. niruri* was purchased from Shubham Enterprises Patna, and its taxonomic authentication was carried out by Plant Taxonomist, Technical Officer Flora at Jharkhand Biodiversity Board, Ranchi, Jharkhand, India. The leaves were removed and washed in tap water, followed by distilled water. Next, the leaves were air-dried for 10 days, powdered, and kept in an airtight container until further downstream application.

### 2.3. Collection of the Pathogenic Bacterial Strain

Two different species of the genus *Xanthomonas*, viz. *X. axonopodis* pv. (BD0001) and *X. campestris* (BH0001), were obtained from Microbial Type Culture Collection (MTCC), The Indian Agriculture Research Institute (IARI), New Delhi, India. 

### 2.4. Isolation of Leaf Extract of P. niruri and Preparation of GS-AgNP-LEPN

The leaf extract of *P. niruri* was obtained via Soxhlet extraction by using a solvent mixture of chloroform, methanol, and water (1:1:1, *v*/*v*/*v*) at 70 °C. The synthesis of GS-AgNP-LEPN was carried out as described previously [55], with a slight modification. Briefly, 10 mL of leaf extract of *P. niruri* was added to 90 mL of 1 mM AgNO_3_ solution, followed by heating in a water bath (set at 80 °C) for 10 min. The resultant reaction mixture (reddish, brown-colored) was centrifuged at 12,000 rpm for 10 min at room temperature. The pellet (containing GS-AgNP-LEPN) obtained was washed three times with deionized water and eventually with acetone. The final pellet was dried and stored for further characterization and downstream analysis.

### 2.5. Nano Tracking Analyzer for Counting GS-AgNP-LEPN 

The concentration and size distribution of GS-AgNP-LEPN were determined using the NanoSight nano tracking analyzer (NTA) instrument and software v3.1 (Malvern Panalytical Ltd., Malvern, UK). The sample was diluted 1000 times, and scanning of the sample was carried out three times for 60 s each. 

### 2.6. Scanning Electron Microscopy 

The morphology and shapes of GS-AgNP-LEPN were analyzed by using FEI Quanta 250 at the Council of Scientific and Industrial Research (CSIR)–Central Drug Research Institute (CDRI), Lucknow, Uttar Pradesh, India. Briefly, a drop of the sonicated aqueous suspension of the GS-AgNP-LEPN sample (minimal amount) was placed on a carbon-coated copper grid; the excess sample was removed with blotting paper. Next, the films on the grid were dried for 5 min under a mercury lamp and then examined with SEM.

### 2.7. UV–Visible Spectroscopy

The characterization of GS-AgNP-LEPN was performed by using a Systronics UV–Visible spectrometer, Model 117 (Systronics India Limited, Ahmedabad, India). The specific absorbance spectrum was obtained by scanning the absorbance of the sample at room temperature in the wavelength range of 300–700 nm at a resolution of 1 nm. 

### 2.8. X-ray Diffraction

The crystallographic properties of GS-AgNP-LEPN were analyzed by using Bruker Alpha X-ray diffraction (XRD), Model No. B8 (at Cytogene Lab, Lucknow, Uttar Pradesh, India). A thin film of the dried samples was dispersed on a glass slide, and the XRD spectrum was obtained as a plot of the intensity of scattered X-rays at diverse angles. A monochromatic Cu Kα radiation instrument with a wavelength (λ) of 1.5406 Å was used with a scan rate of 0.2 s/step (step size 0.02°), fitted with a nickel monochromator at a voltage of 40 kV and a tube current of 30 mA.

### 2.9. Fourier Transform Infrared Spectroscopy

For the identification of biomolecules present in the *P. niruri* leaf extract that could potentially be responsible for reducing Ag^+^ ions, the Fourier transform infrared spectroscopic (FTIR) spectrum was obtained in the wavelength range of 3000–1000 nm by Bruker Alpha FTIR, Model No. ECO-ATR (Karlsruhe, Germany). The KBr pellets were prepared by homogenizing the sample with KBr powder at a mass ratio of 1:100.

### 2.10. High-Performance Liquid Chromatography 

The leaf extract of *P. niruri* was powdered after drying and was kept at 4 °C in an air-tight container away from light and humidity for high-performance liquid chromatography (HPLC) analysis using the HPLC system (Shimadzu available at Cytogene Lab, Lucknow, India). The solvents used were acetonitrile, methanol, and distilled water in the ratio of 70:25:5.

### 2.11. Molecular Docking Study

Three-dimensional atomic coordinates for the crystal structures of FAD-containing ferredoxin-NADP^+^ reductase (PDB id: 4b4d) and type-III effector XopAI (PDB id: 6kly) of *X. axonopodis* pv. Citri were downloaded from the RCSB-Protein Data Bank (PDB). The structures determined by the X-ray diffraction method at a resolution of 1.50 Å and 2.01 Å, respectively, have been previously reported [52,56]. Molecular docking preparation and simulation were performed using UCSF Chimera Version 1.14 and AutoDock 4.2 [57,58,59,60]. UCSF Chimera was loaded with the structure of FAD-containing ferredoxin-NADP reductase and type III effector XopAI for molecular docking preparation. In order to clean and improve protein structures, ligands and other heteroatoms were eliminated. Then, the steepest descent approach with 100 steps (step size 0.02) and the conjugate gradient method with ten steps (step size 0.02) were utilized to minimize the energy of protein structures using UCSF Chimera. The 3D structures of ampicillin and nirurinetin were downloaded from the NCBI-PubChem database. Ampicillin molecules were kept as the reference/control, as they are known antibiotics that have been used for many years, and their use as a control group allows scientists to compare the effects of other treatments to the control group and more accurately determine their effectiveness.

The molecular docking of phytochemical constituents of *P. niruri*, namely, ampicillin, and nirurinetin, was performed with the active site of FAD-containing ferredoxin-NADP^+^ reductase and ADP-Ribosyl transferase domain of the type-III effector XopAI protein to predict the possible mechanism of the antibacterial action. 

Docking was performed near the central cleft of XopAI by keeping the number of points as 46, 52, and 52 in the X, Y, and Z dimensions, and the center grid box values were kept at −6.052, −13.599, and −13.431 for the X-, Y-, and Z-center with 0.375 Å spacing. Docking was performed in the NADP+ binding pocket for FAD-containing ferredoxin-NADP reductase by keeping the number of points as 48, 52, and 48 in the X, Y, and Z dimensions, and the center grid box values were kept at 14.153, 18.923, and 26.71 for the X-, Y-, and Z-center with 0.375 Å spacing. The grid box for the central cleft of XopAI and the NADP+ binding pocket of ferredoxin-NADP reductase provides enough space for the ligands’ translational and rotational walks. The docking was performed with 30 independent runs and a maximum number of 27,000 GA operations generated on a single population of 150 individuals. As the default parameter, the operator weights for the rates of crossover, gene mutation, and elitism were set to 0.80, 0.02, and 1, respectively. 

### 2.12. Isolation and Characterization of APF-EV

APF from LEPN was extracted as described previously [61,62], followed by the isolation of EV from APF-LEPN, as described previously [63]. Briefly, APF is extracted from the leaf by first making a small incision in the leaf to expose the cells. Then, a needle is inserted into the exposed cells, and the apoplast fluid is drawn out. The fluid is then collected in a container for further analysis. EVs are isolated from APF using a centrifugation-based method. The fluid is first centrifuged at a high speed to pellet out larger cellular debris, followed by a low-speed centrifugation step to separate the extracellular vesicles from the remaining fluid. This can be followed by a filtration step to further remove any remaining cellular debris and proteins. Finally, the extracellular vesicles can be purified using a magnetic-activated cell sorting (MACS) technique to isolate them from the remaining solution. The loading of GS-AgNP-LEPN in APF-LEPN-EXO was accomplished as described previously [64]. Briefly, the sonication method was used for loading NPs into EVs using a medium-powered ultrasound device to break down the cell membrane of the EV, allowing the NPs to enter. The size distribution and particle concentration of the APF-EV were determined by the nanotracking analyzer (NTA), and the presence of the marker protein TET8 was determined by the dot-blot analysis.

### 2.13. Antibacterial Assay via the Disc Diffusion Test

Two plant pathogenic microorganisms. viz. *X. axonopodis* pv. and *X. campestris* pv., were used for evaluating the antimicrobial activity of GS-AgNP-LEPN using a disc diffusion test [60]. Whatman No. 1 filter paper was punched uniformly, sterilized, and then impregnated with either an antibiotic (ampicillin) or GS-AgNP-LEPN. Preparation of nutrient agar: 23 g of synthetic nutrient agar medium was dissolved in 1000 mL of distilled water, followed by the addition of a pinch of agar to it. The mixture was boiled and autoclaved at 12 °C for 15 min. Preparation of inoculum: Master suspension up to 10^−5^ was prepared by serial dilution. Inoculation of plates: 1 mL of diluted suspension (10^−5^) was pipetted on a nutrient agar plate and swirled evenly using a cotton swab. Loading of disc: The plates after swabbing were kept at room temperature for 10–15 min to allow the surface moisture to be absorbed; then, the discs were loaded with impregnated discs of either the antibiotic (ampicillin) or GS-AgNP-LEPN through sterilized forceps under laminar airflow. The plates were then incubated for 24 h at 37 °C. 

### 2.14. Western Blot Analysis

The identification and quantification of FAD-FNR, XopAI, tubulin, and GAPDH in the protein samples of the untreated control and different dilutions (0.0625×, 0.25×, and 1×) of nirurinetin-treated *X. axonopodis* pv. were conducted by Western blotting. Briefly, 15 μg protein samples were separated by 8% SDS gel electrophoresis and then transferred to a polyvinylidene difluoride membrane. After incubation with 5% skim milk in tris-buffered saline (TBS) (blocking buffer) for 1 h, the membrane was further incubated with specific primary ABs (1:1000 dilution) in blocking buffer (5% skim milk) overnight at 4 °C, followed by washing three times with TBST and further incubation with goat anti-rabbit immunoglobulin G (IgG) H&L (horseradish peroxidase (HRP)) secondary AB (1:5000 dilution) for 2 h at room temperature. Immunoreactive bands were detected using the enhanced chemiluminescence substrate (Bio-Rad, Hercules, CA, USA) and imaged using the Azure Biosystems (Dublin, CA, USA) Gel Documentation system (C600).

### 2.15. Statistical Analysis

The data are represented as the mean ± standard error mean (SEM) of three independent experiments. The statistical significance was calculated by using the student’s *t*-test, considering * *p* < 0.05 and ** *p* < 0.01.

## 3. Results

### 3.1. Physicochemical Characterization of GS-AgNP-LEPN 

**Figure 1:**
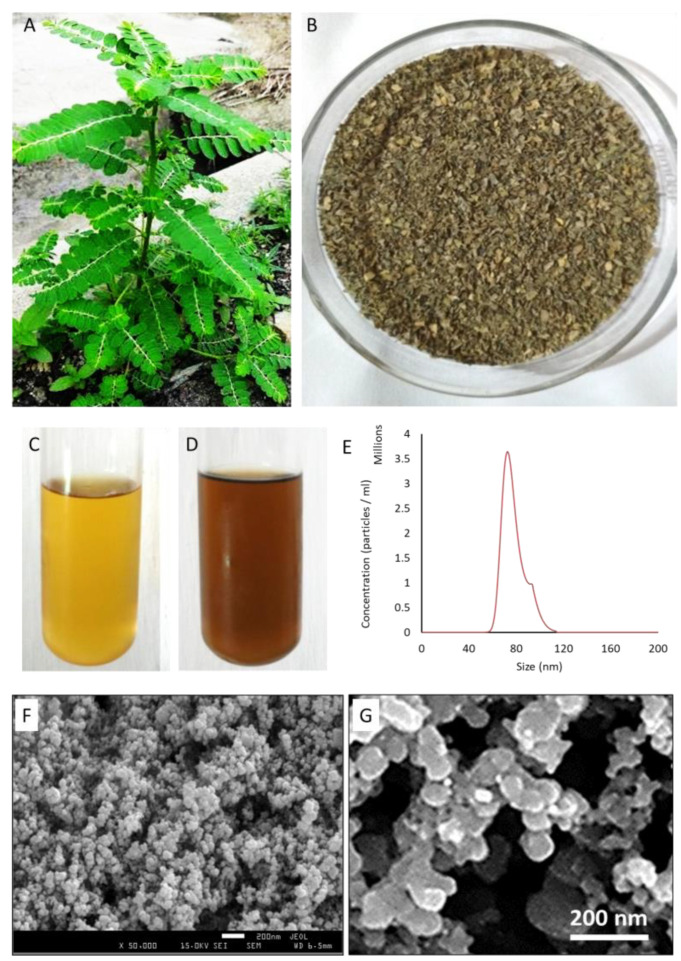
Biosynthesis of GS-AgNP-LEPN. A representative color photograph of (**A**) *P. niruri* plant; (**B**) powdered leaves of *P. niruri*; (**C**) mixture containing leaf extract of *P. niruri* and 1 mM AgNO_3_ in the proportion of 1:3; (**D**) GS-AgNP-LEPN solution after 24 h. Representative (**E**) size distribution by NTA and (**F**) morphology by SEM (Scale bar: 200 nm) of GS-AgNP-LEPN and (**G**) their SEM image at high resolution.

As noted above, there are various advantages of green synthesis compared to the conventional techniques of NP synthesis [32,33,65]. Previously, *P. niruri* has been reported to exert various therapeutic potentials [66]. In this study, we isolated the extracts of powdered leaves of *P. niruri* (Figure 1A,B) via the Soxhlet extraction method. Initially, the leaf extract was light greenish, and the AgNO_3_ solution was a clear solution. Next, the yellow-colored mixture (1:3 ratio) of the solutions of the leaf extract of *P. niruri* and 1 mM AgNO_3_ (Figure 1C) turned into a brown color, demonstrating the formation of GS-AgNP-LEPN (Figure 1D), which is presumably due to the excitation of surface plasmon on nanoparticles [67]. The size distribution analysis of GS-AgNP-LEPN via NTA analysis showed that the NP are mostly less than 100 nm (Figure 1E), and morphological analysis by SEM demonstrated the spherical shape of GS-AgNP-LEPN (Figure 1F,G). The average size of GS-AgNP-LEPN was found to be 40–70 nm, as detected from the SEM analysis, which was again found to be consistent with the size distrubtion data via NTA analysis (Figure 1E–G). 

### 3.2. Analytical Characterization of GS-AgNP-LEPN by UV–Visible Spectroscopy, XRD, and FT-IR

**Figure 2:**
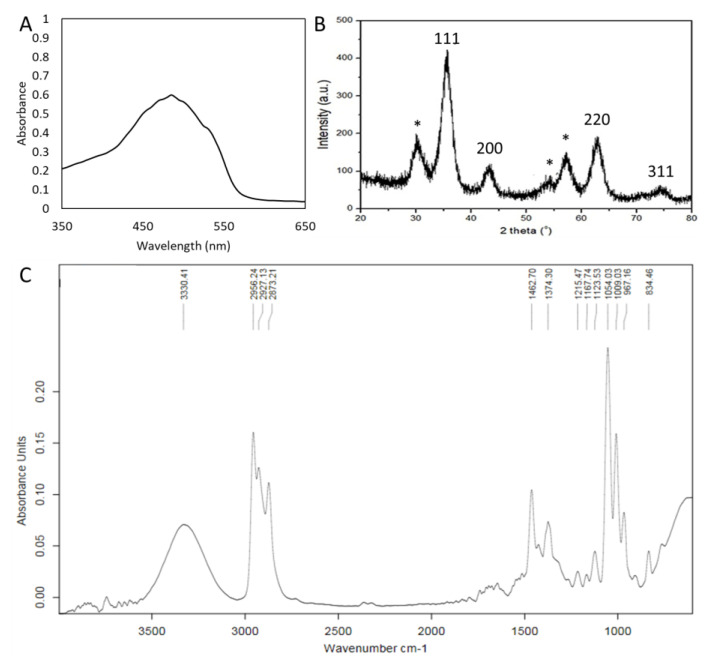
Characterization of GS-AgNP-LEPN. Representative (**A**) spectrum of UV–Visible absorbance in the wavelength range of 350–650 nm, (**B**) XRD spectrum, and (**C**) FT–IR spectrum of GS-AgNP–LEPN.

For the identification of synthesized NP, the characterization of GS-AgNP-LEPN was performed through UV–visible spectroscopy, XRD analysis, and FT-IR analysis (Figure 2A–C). The UV–vis spectrometry analysis of GS-AgNP-LEPN showed maximum absorbance with a characteristic peak at 485 nm (Figure 2A). The AgNPs usually show maximum absorbance around 400 nm, and the shift for a higher wavelength, as occurs in our result, can be attributed to the aggregation of the NPs, as can be seen in the SEM images (Figure 1F,G). XRD analysis of GS-AgNP-LEPN was performed to understand the molecular and crystal structure. The values of 2θ were calculated following Bragg’s law and were found to be around 32.4°, 35°, 41.8°, 54°, 57°, 63°, 71.5°, and 74.9, corresponding to interplanar spacing (d) of 0.28 Å, 0.26 Å, 0.22 Å, 0.17 Å, 0.16 Å, 0.15 Å, 0.13 Å, and 0.13 Å, respectively. The results suggest that GS-AgNP-LEPN is highly crystalline (Figure 2B). Based on previous reports, the XRD-specific peaks for GS-AgNPs-LEPN are mainly due to the (111), (200), (220), and (311) planes. These peaks can be attributed to the crystal structure of silver nanoparticles, which is known to be face-centered cubic. The peak (111) is due to the (111) plane of the face-centered cubic structure. The intensity of this peak is very high, as the (111) plane has the highest density of atoms. Regarding (200), the peak is due to the (200) plane of the face-centered cubic structure. This peak is usually less intense than the (111) peak. Regarding (220), the peak is due to the (220) plane of the face-centered cubic structure. This peak is usually less intense than the (111) and (200) peaks. Regarding (311), the peak is due to the (311) plane of the face-centered cubic structure. This peak is usually the least intense of the four peaks, which is in accordance with the previous reports. X-ray diffraction results clearly show that the AgNPs formed by the reduction of Ag+ ions by the LEPN extract are crystalline in nature. The unassigned peaks at 2θ = 32.4°, 57°, and 63°, denoted by (*) in Figure 2B, are thought to be related to crystalline and amorphous organic phases [68,69]. The FT-IR characterization of GS-AgNP-LEPN showed peaks at the wavenumbers 3330.41, 2956.24, 2927.13, 2873.21, 1462.61, 1374.40, 1215.47, 1167.74, 1123.53, 1054.03, 1009.03, 967.16, and 834.46 cm^−1^ (Figure 2C). Based on the previous literature, the FT-IR spectra of GS-AgNPs are typically characterized by several distinct peaks. These peaks are attributed to certain functional groups present in the capping agent molecule used to stabilize the nanoparticles. Here, the LEPN is acting as a capping agent, which is employed to stabilize the synthesis of GS-AgNP-LEPN. It also facilitates the reduction of silver ions to AgNP. The most common peaks observed in the FT-IR spectra of AgNPs are 3400–3100 cm^−1^, 1640–1400 cm^−1^, 1220–1100 cm^−1^, and 800–700 cm^−1^. The peak between 3400 and 3100 cm^−1^ is attributed to the presence of O–H stretching and N-H bending vibrations of alcohols, phenols, and amines present in the capping agent. The peak between 1640 and 1400 cm^−1^ is attributed to the presence of C=O stretching vibrations of carboxylic acids, amides, and esters present in the capping agent. The peak between 1220 and 1100 cm^−1^ is attributed to C-N stretching vibrations of amines and amides present in the capping agent. The peak between 800 and 700 cm^−1^ is attributed to the presence of C-H bending vibrations of aromatic and aliphatic hydrocarbons present in the capping agent [70,71].

### 3.3. HPLC Analysis of the Leaf Extract of P. niruri (LEPN) Showed Active Constituents

**Figure 3:**
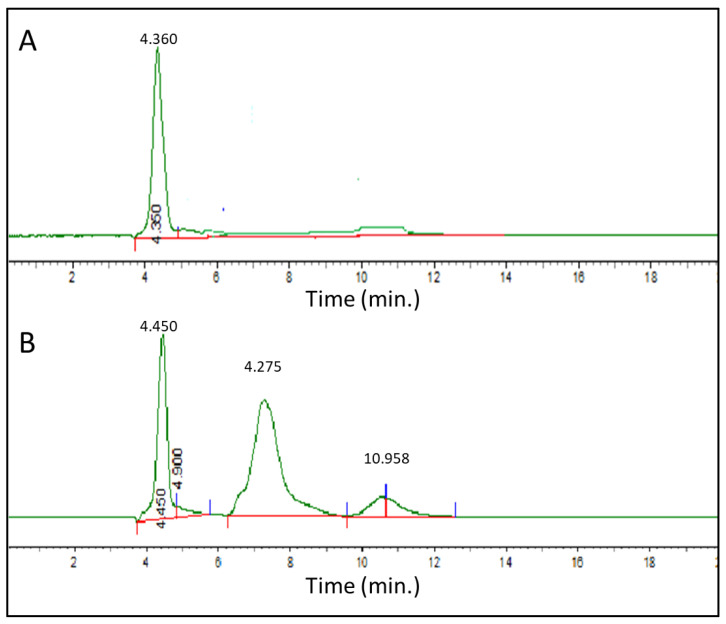
HPLC analysis of LEPN. Representative HPLC chromatograms of (**A**) standard phyllanthin, showing a peak at the retention time of 4.36 min, and (**B**) leaf extract of *P. niruri*, displaying a peak at the retention time of 4.450 min, with two additional peaks at 7.275 min and 10.958 min.

To understand which chemical constituents are potentially responsible for the biological activity, the quantitative analysis of LEPN was performed by using HPLC. Previous studies have reported that phyllanthin is one of the primary chemical constituents with biological activity [72,73,74]. Therefore, we analyzed LEPN by HPLC and compared it with that of a standard phyllanthin. Our HPLC results showed a symmetrical peak for standard phyllanthin at the retention time of 4.36 min (Figure 3A). Three other peaks were found: one at 4.450 min corresponding to phyllanthin and two additional peaks at 7.275 min and 10.958 min, suggesting the presence of other constituents (Figure 3B). Of the two additional peaks, the peak around the retention time of 11 could be due to nirurinetin based on the comparison with a previous report [75].

### 3.4. Nirurinetin Could Be Binding with FAD-Containing Ferredoxin-NADP Reductase and Type-III Effector XopAI Protein

#### 3.4.1. Docking Studies with FAD-Containing Ferredoxin-NADP^+^ Reductase

**Figure 4:**
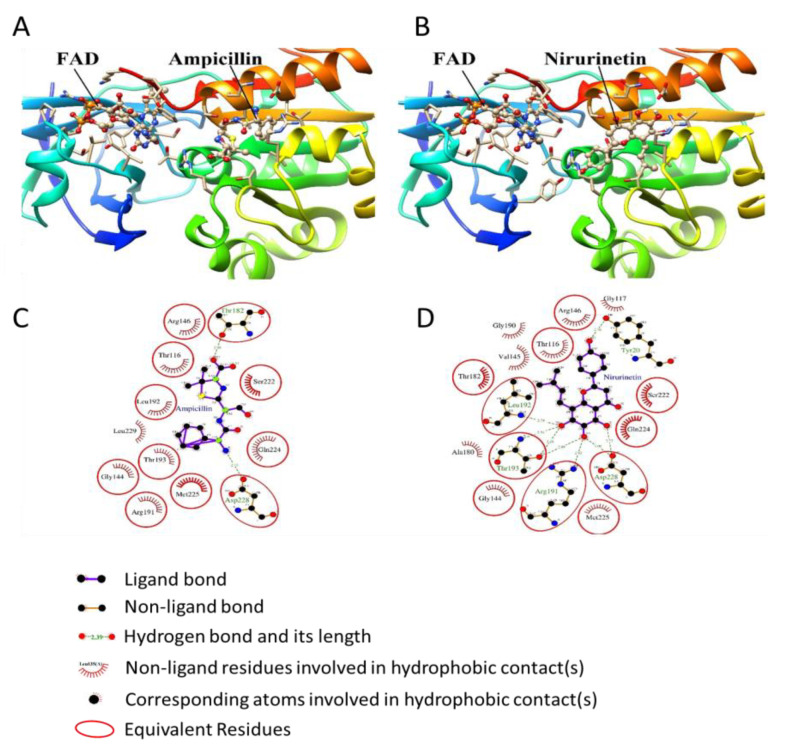
Molecular docking analysis of ampicillin and nirurinetin with FAD-containing ferredoxin-NADP reductase. (**A**,**B**) The binding pattern of (**A**) ampicillin and (**B**) nirurinetin with an FAD-containing ferredoxin-NADP reductase active site pocket. Ligands are represented in the ball & stick model and active site pocket of NADP reductase in the surface & ribbon structure. Nirurinetin exhibited a binding pattern like ampicillin. (**C**,**D**) Assessment of H-bonding and hydrophobic interaction pattern of (**C**) ampicillin and (**D**) nirurinetin with FAD-containing ferredoxin-NADP reductase active site pocket.

The ferredoxin-NADP+ reductase of *X. axonopodis* pv. citri plays a vital role in maintaining the infection. Hence, its inhibition efficiently attenuates the bacterial response [12,52,53]. The docking scores of NADP, ampicillin, phyllanthin, nirurinetin, and rutinoside are provided in Table 1. The molecular docking results revealed that ampicillin bound to the NADP^+^ binding pocket of ferredoxin-NADP+ reductase and established H-bonding with Asp228, Arg146, Thr182, Ser222, Gly117, Thr116, Thr193, and Tyr20 residues. Phyllanthin, nirurinetin, and rutinoside are also bound to the NADP^+^ binding pocket of ferredoxin-NADP+ reductase (Figure 4A,B), like NADP and ampicillin. Among these, nirurinetin exhibited the highest affinity for the NADP^+^ binding pocket, because it bound with the lowest binding energy (−10.32 kcal/mol) (Table 1). Phyllanthin, nirurinetin, and rutinoside exhibited similar H-bonding and hydrophobic interaction patterns to those shown by ampicillin (Figure 4C,D).

#### 3.4.2. Docking Studies with Type-III Effector XopAI Protein

Molecular docking studies were also performed to explore the antibacterial phytochemicals of *P. niruri* (namely, phyllanthin, nirurinetin, and rutinoside) against the ADP-ribosyl transferase of type-III effector XopAI protein, which has been found to play a role in the survival and virulence of *X. axonopodis* pv. [54]. The docking scores of ligands were compared with ampicillin as a reference molecule. The molecular docking results of phyllanthin, nirurinetin, and rutinoside and ampicillin with Type III Effector XopAI protein are summarized in Table 2. According to the PDB structure, as posited by Liu et al. [56], the central cleft of XopAI is an Arg peptide-binding cleft located between the N- and C-lobes; this appears to be the active site. The key residues of the central cleft are Trp154, Ala228, Arg260, Glu263, and Glu265. Specific arginine recognition is mediated by H-bonding with Trp154, Thr155, and Thr156 residues present in the backbone, as well as with a Glu265 residue of the side chain.

**Figure 5:**
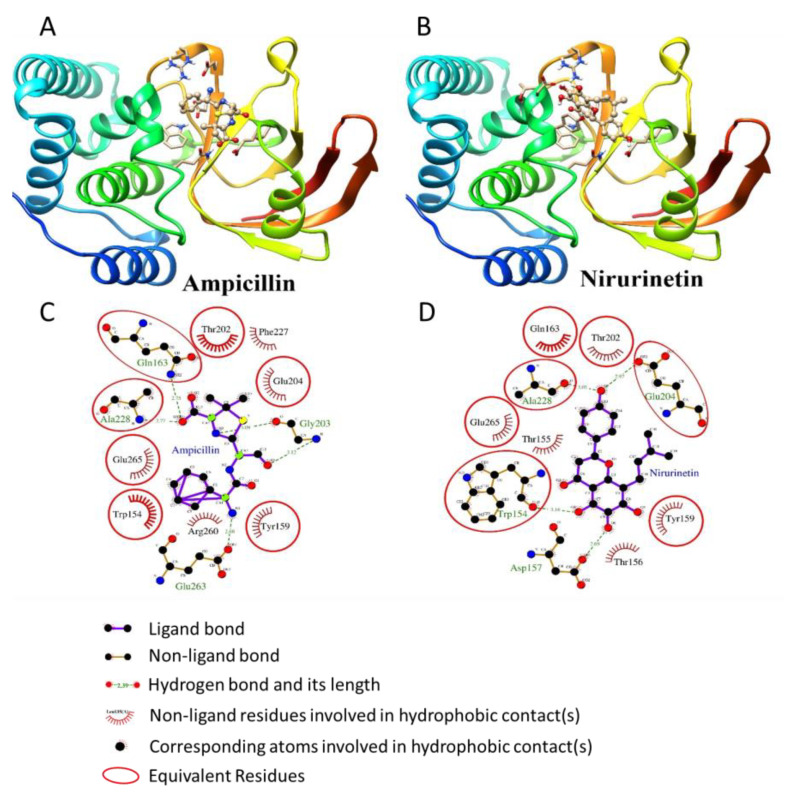
Molecular docking analysis of ampicillin and nirurinetin with type-III Effector XopAI Protein. (**A**,**B**) The binding pattern of (**A**) ampicillin and (**B**) nirurinetin with Arg-peptide-binding cleft (central cleft) of type III effector XopAI. Ligands are represented in the ball & stick model and type III effector XopAI in a ribbon structure. Nirurinetin exhibited a similar binding pattern to that shown by the ampicillin molecule. (**C**,**D**) Assessment of H-bonding and hydrophobic interaction pattern of (**C**) ampicillin and (**D**) nirurinetin with Arg peptide-binding cleft (central cleft) of type III effector XopAI.

Our molecular docking simulation study demonstrated that ampicillin established H-bonds with Gln163, Ala228, Glu263, Arg260, and Gly203 residues of the central cleft. Phyllanthin, nirurinetin, and rutinoside bound to the central cleft of XopAI, as did ampicillin (Figure 5A,B). Nirurinetin bound to the Arg peptide-binding cleft with lower binding energy (−6.13 kcal/mol) compared to ampicillin (−6.00 kcal/mol) and hence displayed higher affinity toward the central cleft of XopAI (Table 2). All phytochemicals exhibited similar H-bonding and hydrophobic interactions to those shown by ampicillin (Figure 5C,D). 

### 3.5. APF-EV Loaded with GS-AgNP-LEPN Showed Enhanced Antimicrobial Activity towards X. axonopodis pv. via Nirurinetin-Dependent Inhibition of FAD-FNR and XopAI

**Figure 6:**
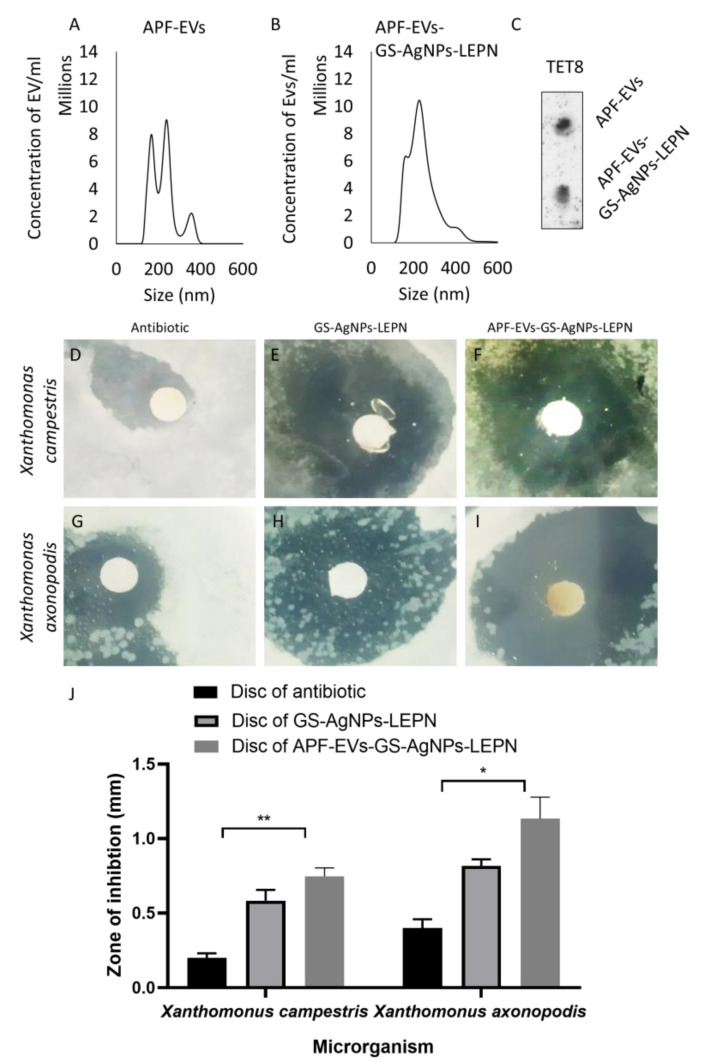
Comparing the antimicrobial activity of APF-EV-GS-AgNP-LEPN and GS-AgNP-LEPN against two different *Xanthomonas* species by a disc diffusion test. Representative (**A**,**B**) size distribution and (**C**) immunoblot showing the expression of TET8 in protein samples of APF-EV and APF-EV-GS-AgNP-LEPN. Representative zone of inhibition of Ampicillin, GS-AgNP-LEPN, and APF-EV-GS-AgNP-LEPN against (**D**–**F**) *Xanthomonas campestris* and (**G**–**I**) *Xanthomonas axonopodis* pv. (**J**) Representative bar graph showing the zone of inhibition by Ampicillin and GS-AgNP-LEPN. Data in (**J**) are presented as the mean ± SEM (N = 3). Disc of antibiotic vs. Disc of GS-AgNP-LEPN, Disc of antibiotic vs. Disc of APF-EV-GS-AgNP-LEPN; * *p* < 0.05 and ** *p* < 0.01.

As the coating of NP with a bio-membrane enhances the biocompatibility of NP [76], the presence of leaderless proteins in the APF-EV represents unconventional protein secretion (UPS) pathways associated with cell wall remodeling and resistance to infection [77,78]. Therefore, we utilized the APF of LEPN for the isolation of EV and APF-EV-GS-AgNP-LEPN. After that, we examined the size distribution and the expression of the TET8 protein (a common marker for plant-derived EV) in APF-EV before and after the encapsulation of GS-AgNP-LEPN, namely, APF-EV and APF-EV-GS-AgNP-LEPN, respectively. The overall range of the size distribution of APF-EV was found to shift from 117.5–407.5 nm for the APF-EV to 106.5–508.5 nm for APF-EV-GS-AgNP-LEPN. Additionally, the concentration of particles/mL was slightly augmented after the loading (Figure 6A,B). Further, the examination of the variation in the size range vs. the percentile of EVs demonstrates that the majority of APF-EVs-GS-AgNPs-LEPN have a bigger size range as compared to the APF-EVs (Supplementary Figure S1).

The presence of the TET8 protein, as determined by the dot-blot, established that the isolated vesicles were indeed plant-derived EV, and they were not significantly affected by the encapsulation of GS-AgNP-LEPN (Figure 6C), signifying the persistent structural stability of APF-EV before and after encapsulation.

The antibacterial activity of GS-AgNP-LEPN, APF-EV- GS-AgNP-LEPN, and the antibiotic (ampicillin) was evaluated against two species of *Xanthomonas* (namely, *X. campestris* and *X. axonopodis* pv.) by using a disc diffusion test. Briefly, a sterilized disc was dipped separately in 12 µM of ampicillin and GS-AgNP-LEPN and APF-EV-GS-AgNP-LEPN, followed by the measurement of the zone of inhibition (Figure 6 D–J). The zone of inhibition was more significant in the APF-EV-GS-AgNP-LEPN- and GS-AgNP-LEPN- treated *X. campestris* as compared to that treated with ampicillin (Figure 6D–F,J). Similarly, the zone of inhibition was more significant in the APF-EV-GS-AgNP-LEPN- and GS-AgNP-LEPN- treated *X. axonopodis* pv. as compared to that treated with ampicillin (Figure 6G–J). Conclusively, the APF-EV-GS-AgNP-LEPN and GS-AgNP-LEPN possess significant antibacterial activity towards both *Xanthomonas* species. Notably, the antibacterial potential of APF-EV-GS-AgNP-LEPN was found to be more than that of GS-AgNP-LEPN. Our results, for the first time, demonstrated that APF-EV-GS-AgNP-LEPN and GS-AgNP-LEPN inhibit the growth of *X. axonopodis* pv., indicating its potential as a treatment for citrus canker. 

**Figure 7:**
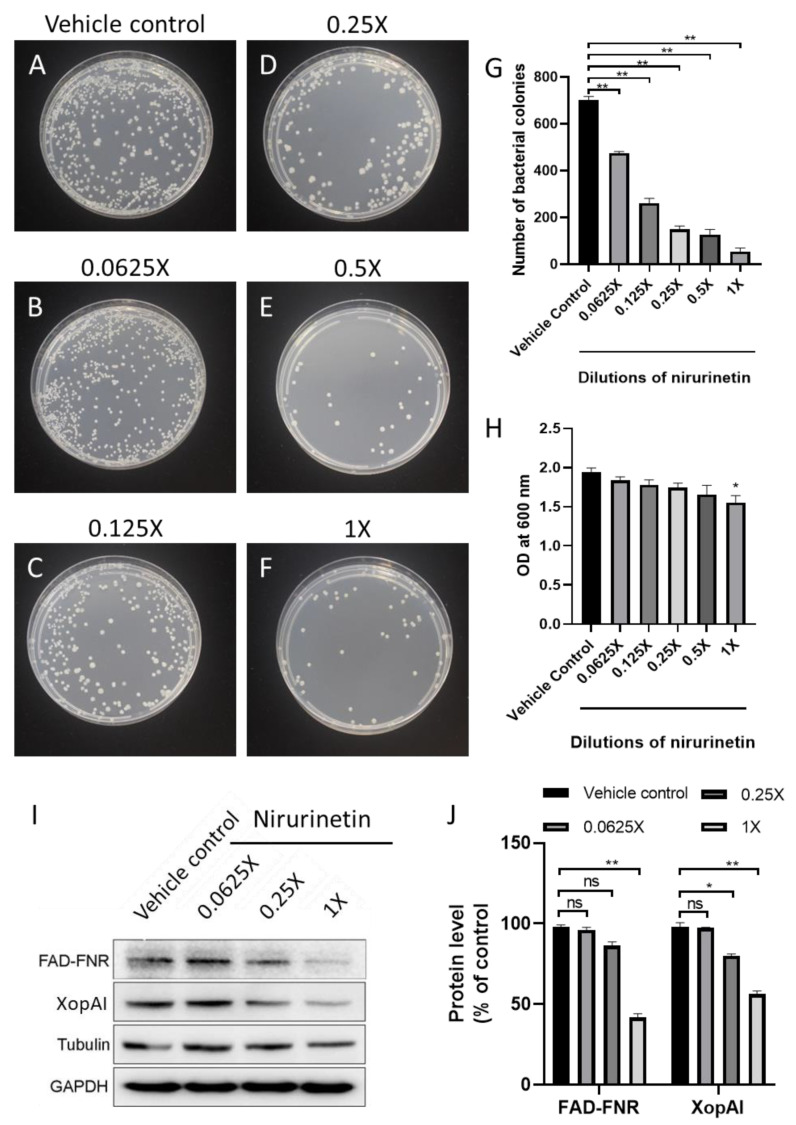
Effect of nirurinetin on the protein level of FAD-FNR and XopAI in *X. axonopodis* and its bacterial growth. (**A**–**H**) Representative images showing the effect of different dilutions of nirurinetin on the growth of *X. axonopodis* pv. and their quantitative bar graphs. (**I**,**J**) Representative immunoblots showing the effect of different dilutions of nirurinetin on the growth of *X. axonopodis* pv. and their quantitative bar graphs. Data in (**G**,**H**,**J**) are presented as the mean ± SEM (N = 3). Vehicle control vs. different concentrations of nirurinetin; * *p* < 0.05, ** *p* < 0.01, ns = not significant.

To determine whether the antibacterial activity we found is indeed through the effect of nirurinetin on the FAD-FNR and XopAI of *X. axonopodis* pv., Western blot analysis was conducted on the protein samples of *X. axonopodis* pv. treated with different concentrations of nirurinetin and compared with the vehicle-treated group. The treatment of nirurinetin significantly reduced bacterial growth (Figure 7A–H) and reduced the protein level of FAD-FNR and XopAI (Figure 7I,J), signifying that nirurinetin is the key active constituent of APF-EV-GS-AgNP-LEPN and GS-AgNP-LEPN in its effect on *X. axonopodis* pv.

## 4. Discussion

Citrus canker is a highly infectious plant disease caused by different variants of the bacterium *X. axonopodis* pv. in the field crops of citrus species including lemon, orange, and grapefruit. The bacteria propagate through lesions in the stem, leaf, and fruit. Wind-driven rain is the leading dispersing agent of the bacterium, while water congestion in the leaves leads to the penetration of the bacterium into the host plant species through stomata or wounds [78]. Severe infections lead to defoliation, premature fruit drop, blemished fruit, twig die-back, and weakening of the tree, resulting in great economic losses. Infection also readily spreads among recently planted crops. The current strategies for eliminating citrus canker include exclusion, eradication (uprooting and burning), sanitation (safe practices), and copper treatment. However, the current methods are time-consuming, require intensive labor, and incur additional expenditures [19]. Better alternatives are needed.

*X. axonopodis* pv. citri is a Gram-negative, rod-shaped bacterium characterized by host-specific pathogenicity. Several proteins are responsible for achieving the virulence of *X. axonopodis* pv. (Table 2). For most pathogenic bacteria, biofilm on the host protects the bacterium against environmental stress and helps it resist t host defense responses. *X. axonopodis* pv. citri is no exception; it requires biofilm over the host surface to achieve maximum virulence. The biofilm proteome is composed of several membrane proteins and receptor or transport proteins which play a major role in maintaining cellular signaling and homeostasis within the host [79]. Much of the Gram-negative bacteria employ a type-III-secretion system (T3SS) to deliver effector proteins in the host cytoplasm, thereby suppressing pathogen-associated molecular pattern (PAMP)-triggered immunity or host resistance proteins recognition [80,81]. However, protein ADP-ribosylation, a post-translational modification, has recently emerged as a critical player in launching an effective defense mechanism by bacteria and stealthily associating the cellular process of the host [82]. The effector proteins XopAI and HopO1 share a VIP2 ADP-ribosylation motif which helps in translating protein toxins into the host cellular machinery [83,84]. Moreover, the host plant employs an in-house defense mechanism against pathogens by producing reactive oxygen species (ROS) which pathogens need to overcome to maintain infection. In contrast, FNRs are redox flavoenzymes that help *X. axonopodis* pv. combat such plant defense mechanisms. *X. axonapodis* pv. citri has a gene (fpr) responsible for encoding FAD-FNR to survive the alternating levels of ROS in the host plant [12]. Therefore, our study evaluated FAD-FNR and XopAI as key players in the antimicrobial activity against *X. axonopodis* pv. and, thus, as potential targets for treatments. 

NP-DDS is quite an effective method in nanobiotechnology; it has led to the creation of several antimicrobial drugs as alternatives to conventional antibiotics. Properties such as a longer retention time, the promotion of local antimicrobial activity, and drug-free virulence make NP-DDS attractive as a means to combat bacterial infection, particularly in the face of rising antibiotic resistance [85]. Our approach was not only effective but also environmentally friendly. We synthesized NP with a green method and utilized a plant extract in combination with NP. We chose *P. niruri* because it has been reported to have beneficial properties in terms of antimicrobial activity [43,86,87,88]. We synthesized GS-AgNP-LEPN with the leaf extract of *P. niruri* as the reducing agent for AgNP.

The interaction between nanomaterials and biological membranes has long been studied owing to the advantages rendered to DDS. Recently, EV have attracted considerable attention as a drug delivery tool due to their excellent biocompatibility, low immunogenicity, and high circulation [36]. The use of plant-derived EV for theranostic purposes, however, has not been investigated much. Considering the excellent properties of EV, we, for the first time, isolated APF-EV from the leaves of *P. niruri* and encapsulated GS-AgNP-LEPN. This system showed good antibacterial activity against *X. axonopodis* pv., the bacterium responsible for citrus canker. 

*P. niruri* leaves contain a wide variety of phytochemicals. The major significant phytoconstituents extracted from *P.niruri* are niruretin, nirurrine, nirurin, phyllanthin, and phyllanththenol. From our HPLC measurements of GS-AgNP-LEPN, we determined phyllanthin and nirurinetin to be active antimicrobial constituents. Therefore, we proceeded with phyllanthin and nirurinetin to target the ADP-ribosylation motif of the effector protein XopAI of *X. axonopodis* pv.

To establish a potential mechanism underlying the antimicrobial effect of our proposed delivery system, a molecular docking approach was employed. The results showed that nirurinetin could bind to FAD-FNR and XopAI with augmented binding energies (−10.32 kcal/mol and −6.13 kcal/mol, respectively) in comparison to phyllanthin (−6.42 kcal/mol and −2.93 kcal/mol, respectively). This strongly indicated that GS-AgNP-LEPN could be an effective treatment for citrus canker and that this efficacy was due to nirurinetin targeting the FAD-FNR and XopAI of *X. axonopodis* pv. It is quite interesting to note that, although the nirurinetin could be a potential active compound responsible for the antibacterial activity, the AgNPs can further increase the antibacterial effect of the active compounds by providing a larger surface area for the active compounds to interact with. Silver nanoparticles can also help to protect the active compounds from being broken down or degraded by the environment, thus increasing their effectiveness.

In the disc diffusion test, the antimicrobial activity of GS-AgNP-LEPN, compared with the conventional antibiotic ampicillin, showed a significant change in the zone of inhibition, suggesting that the combination of silver and phytoconstituents was particularly effective against citrus canker. However, more exploration is needed to determine whether the silver, the phytoconstituents, or the combination were responsible for the antimicrobial effect. Additionally, the effect of GS-AgNP-LEPN activity in a host–pathogen interactive model could give more insights into the mechanism of uptake and a greater understanding of the cellular process involved in the virulence of *X. axonopodis* pv. In-vitro modeling targeting FNRs should also be explored, as FNRs are another strategy employed by the pathogen in the host. We have demonstrated the potential novel mechanism behind the antimicrobial activity of GS-AgNP-LEPN against *X. axonopodis* pv. via the in vitro and in silico molecular docking studies and present an alternate treatment strategy for combatting citrus canker. Further work includes experimentation with the formulation of APF-EV-GS-AgNP-LEPN GS-AgNP-LEPN as a solution or fertilizer to be used in crop management.

## 5. Conclusions

This is the first study showing that the growth of the bacterium causing citrus canker, *X. axonopodis* pv., is inhibited significantly by a hybrid of APF-EV encapsulating GS-NP, APF-EV-GS-AgNP-LEPN, and a metal NP-based system, GS-AgNP-LEPN. GS-AgNP-LEPN was developed by a green synthesis method and then characterized in terms of size distribution by NTA, morphology by SEM, UV–visible spectroscopy, XRD, and FTIR. APF-EV-GS-AgNP-LEPN and GS-AgNP-LEPN showed significant antimicrobial activity against *Xanthomonas* species (*X. axonopodis* pv., *X. campestris* pv.). The analytical investigation via HPLC revealed the presence of nirurinetin, in addition to phyllanthin, as the major active constituent potentially primarily responsible for the antimicrobial activity.

Two proteins, FAD-FNR and type-III-XopAI, were found to play an important role in the virulence and maintenance of *X. axonopodis* pv., suggesting that these proteins could be used as targets for inhibiting *X. axonopodis* pv. Interestingly, our molecular study revealed that nirurinetin interacted with FAD-FNR and type-III-XopAI with higher binding energy as compared to phyllanthin. The result was further validated by the Western blot, which showed that the inhibition of FAD-FNR and type-III-XopAI by nirurinetin reduced the growth of *X. axonopodis* pv. Therefore, this study not only studied the application of APF-EV-GS-AgNP-LEPN and GS-AgNP-LEPN on *X. axonopodis* pv. for the first time but also proposed a potential mechanism of action for therapeutics of citrus canker via the nirurinetin-dependent inhibition of FAD-FNR and type-III-XopAI.

## Figures and Tables

**Table 1:** Docking results of ligands with ferredoxin-NADP^+^ reductase of *Xanthomonas axonopodis* pv.

Compound Name	Binding Energy (kcal/mol)	Inhibition Constant (K_i_)	Amino Acid Residues Forming H-Bonding with Ferredoxin-NADP^+^ Reductase
Ampicillin	−7.19	5.33 uM	Asp228, Arg146, Thr182, Ser222, Gly117, Thr116, Thr193, Tyr20
NADP	+2.83	-	Arg191, Met225, Thr118, Val145, Arg146, Thr193, Ala180, Thr116, Ser222, Gln224, Thr182, Leu192
Phyllanthin	−6.41	20.11 uM	Tyr20, Asp228, Arg146, Arg191, Leu192
Nirurinetin	−10.32	27.32 nM	Thr193, Asp228, Arg146
Rutinoside	−5.98	41.58 uM	Thr193, Asp228, Thr182, Ser222, Leu192, Met225, Arg146, Thr116

**Table 2:** Docking results of ligands with type-III effector XopAI.

Compound Name	Binding Energy (kcal/mol)	Inhibition Constant (K_i_)	Amino Acid Residues Forming H-Bonding with Type III Effector XopAI
Ampicillin	−6.00	40.23 uM	Gln163, Ala228, Glu263, Arg260, Gly203
Phyllanthin	−2.93	7.17 mM	Gln163, Arg260, Asp157, Glu265, Ala228, Gly203, Glu204
Nirurinetin	−6.13	32.38 uM	Gln163, Glu204, Thr155, Asp157, Trp154, Ala228, Thr156, Gly203
Rutinoside	−4.85	278.97 uM	Gly203, Trp154, Arg260, Glu265, Gln163, Ala228, Glu204, Asp157, Thr156, Thr155

## Data Availability

The data supporting the figures in the manuscript will be available upon reasonable request from the corresponding authors.

## Supplementary Materials

The following supporting information can be downloaded at: https://www.mdpi.com/article/10.3390/nano13081306/s1, Figure S1: A representative graph showing the percentile of EVs in accordance with their size range. The majority of APF-EVs-GS-AgNPs-LEPN are shown have bigger size range as compared to the APF-EVs.; Table S1: Proteins play a role in the virulence of *Xanthomonas axonopodis* pv.; Table S2: Studies related to nanoparticles for the potential treatment of citrus canker and bacterial blight; Table S3: Antimicrobial effects of various species of *Phyllanthus* against various microorganisms. Reference [89] was cited in Supplementary Materials.

## Abbreviations

GS-AgNP-LEPN: Green-synthesized silver nanoparticles with leaf extract of Phyllanthus niruri; EVs: Extracellular vesicles; APF: Apoplastic fluid; APF-EV-GS-AgNP-LEPN: Apoplastic fluid-derived extracellular vesicles encapsulating green-synthesized silver nanoparticles with leaf extract of Phyllanthus niruri; FAD-FNR: Ferredoxin-NADP+ reductase; NP: Nanoparticles; MNP: Metallic nanoparticle; KBr: Potassium Bromide; NTA: NanoSight nano tracking analyzer; FTIR: Fourier transform infrared spectroscopic; HPLC: High-performance liquid chromatography; MACS: Magnetic-activated cell sorting; ROS: Reactive oxygen species.
