# Supplementary material for: Delivery of Apoplastic Extracellular Vesicles Encapsulating Green-Synthesized Silver Nanoparticles to Treat Citrus Canker
Source: Nanomaterials (Basel). 2023 Apr 7;13(8):1306. doi: 10.3390/nano13081306 (PMC10146377; doi:10.3390/nano13081306)
Supplement: Supplementary file 1 [file nanomaterials-13-01306-s001.zip › nanomaterials-2172201-supplementary.pdf]

nanomaterials

*Supplementary Materials*

# Delivery of Apoplastic Extracellular Vesicles Encapsulating Green-Synthesized Silver Nanoparticles to Treat Citrus Canker

**Isha Gaurav [1,†], Abhimanyu Thakur [2,*,†], Gaurav Kumar [3], Qin Long [4], Kui Zhang [2], Rakesh Kumar Sidu [5], Sudha Thakur [6], Rajesh Kumar Sarkar [7], Anoop Kumar [8], Ashok Iyaswamy [1,9,10,*], and Zhijun Yang [1,11,*]**

1 School of Chinese Medicine, Hong Kong Baptist University, Hong Kong SAR 999077 , China
2 Ben May Department for Cancer Research, Pritzker School of Molecular Engineering, University of Chicago, Chicago, IL 60637, USA
3 Clinical Research Division, Department of Biosciences, School of Basic and Applied Sciences, Galgotias University, Greater Noida 203201, Uttar Pradesh
4 Citrus Research Institute, Southwest University, Chinese Academy of Agricultural Sciences, National Citrus Engineering Research Center, Chongqing 400712, China
5 School of Biomedical Engineering, Indian Institute of Technology, Banaras Hindu University, Varanasi 221005, Uttar Pradesh, India
6 National Institute for Locomotor Disabilities (Divyangjan), Kolkata 700090, India
7 Department of Medicine, Division of Biological Sciences, University of Chicago, Chicago, IL 60637, USA
8 Department of Pharmacology, Delhi Pharmaceutical Sciences and Research University (DPSRU), New Delhi 110017, India
9 Mr. & Mrs. Ko Chi-Ming Centre for Parkinson's Disease Research, School of Chinese Medicine, Hong Kong Baptist University, Hong Kong SAR 999077, China
10 Department of Biochemistry, Karpagam Academy of Higher Education, Coimbatore 641021, India
11 Changshu Research Institute, Hong Kong Baptist University, Changshu Economic and Technological Development (CETD) Zone, Changshu 215500, Jiangsu Province, China
* Correspondence: abithakur1211@gmail.com (A.T.); iashok@hkbu.edu.hk (A.I.); yzhijun@hkbu.edu.hk (Z.Y.)
† These authors contributed equally to this work.

**Table S1.** Proteins play a role in the virulence of *Xanthomonas axonopodis* pv.

| Proteins in *X. axonopodis* pv. | Function(s) | Ref. |
|---|---|---|
| Harpin protein; Hpa1 | Elicits a defense response in non-host plants and, to a lesser extent, in host plants.<br><br>Alters the leaf mesophyll structure to allow bacterial cells to aggregate, thereby increasing pathogen virulence. | [1] |
| Bacterial effector protein; AvrXacE2 | Involved in lesion-stimulating disease 1-mediated cell death. | [2] |
| HrpG and HrpX | Coordinates the expression of multiple virulence factors for modification and adaption of the host environment during infection. | [3] |
| hrpB, hrpD, and hrpF | Facilitates pathogenicity in hosts and induces hypersensitivity in nonhost plants. | [4] |
| XacPNP | Enables a plant pathogen to modify host responses to create conditions favorable to its own survival | [5] |

| | | |
|---|---|---|
| fliC (flagellin) | Involved in flagellum-mediated pathogenicity | [6] |
| xpsD | Involved in cellulose degradation | [7] |
| XackatE | Facilitates bacterial colonization and survival in the citrus plant | [8] |
| Xac-LOV | Enhances bacterial motility, exopolysaccharide production, biofilm formation, and adhesion to biotic or abiotic surfaces | [9] |

**Table S2.** Studies related to nanoparticles for the potential treatment of citrus canker and bacterial blight.

| Nanoparticles | Microorganism | Disease | Ref. |
|---|---|---|---|
| Copper composites core-shell copper (CS-Cu), multivalent copper (MV-Cu), and fixed quaternary ammonium copper (FQ-Cu) | Copper-sensitive *X. perforans* strain | Copper-tolerant *X. perforans* and tomato bacterial spot | [10] |
| Nanoscale formulations of $TiO_2$ | *Xanthomonas perforans* | Bacterial spot of tomato | [11] |
| DNA-directed silver NP on graphene oxide (Ag-dsDNA-GO) | *Xanthomonas perforans* | Bacterial spot of tomato in the greenhouse | [12] |
| Nano-Magnesium Oxide | Copper-tolerant *Xanthomonas perforans* | Bacterial spot of tomato | [13] |
| Nano-zinc oxide materials, plate-like Zinkicide SG4 and particulate Zinkicide SG6 | *Xanthomonas citri* | Citrus canker | [14] |
| ZnO/Nano copper composite; nanocopper-loaded silica gel (ZnO-nCuSi) | X*anthomonas 2lfalfa* subsp. *Citrumeloni s* strain F1 | Citrus canker | [15] |
| | *Pseudomonas syringae* pv. *Syringae* | Bacterial spot in other species, e.g., lilac, almond, apricots, peaches, wild beans | |
| | *Clavibacter michiganensis* subsp. *Mic higanensis* | Bacterial wilt and canker in tomato | |

| | | | |
|---|---|---|---|
| Copper/Zinc formulation | Copper tolerance and zinc sensitivity of Mexican strains of *Xanthomonas campestris* pv. *Vesicatoria* | Bacterial spot of pepper | [16] |

**Table S3.** Antimicrobial effects of various species of *Phyllanthus* against various microorganisms.

| Species of Phyllanthus | Microorganisms | Ref. |
|---|---|---|
| *Phyllanthus amarus* | *Aeromonas hydrophila* | [17] |
| | *Escherichia coli, Salmonella typhimurium, Serratia marcescens, Pseudomonas aeruginosa* | [18] |
| | *Shigella dysenteriae, Staphylococcus aureus, Streptococcus pneumoniae, Escherichia coli, Bacillus subtilis, Salmonella typhimurium, Vibrio cholerae, Pseudomonas aeruginosa, Klebsiella pneumoniae* | [19] |
| *Phyllanthus niruri* | *Solanum nigrum, Streptococcus sanguis, Streptococcus salivarius, Streptococcus oralis, Streptococcus mutans* | [20] |
| | *Bacillus cereus, B. subtilis and Staphylococcus aureus Escherichia coli, Proteus rettgeri, Pseudomonas aeruginosa* | [21] |
| | *Escherichia coli, Pseudomonas aeruginosa, Klebsiella pneumoniae, Proteus mirabilis, Salmonella typhyimurium, Shigella flexneri, Staphylococcus aureus, Streptococcus viridian, Candida albicans, Aspergillus flavus and Aspergillus niger.* | [22] |
| | *Escherichia coli, Staphylococcus aureus, Salmonella typhi, Pseudomonas aeruginosa, Klebsiella aerogenes* | [23] |
| | *Candida albicans, Aspergillus niger, Escherichia coli, Bacillus subtilis, Staphylococcus aureus* | [24] |
| | *Staphylococcus aureu, Streptococcus agalactiae* | [25] |
| *Phyllanthus wightianus* | *Bacillus subtilis, Streptococcus pneumoniae, Staphylococcus epidermidis, Proteus vulgaris, Pseudomonas aeruginosa, Klebsiella pneumoniae, Salmonella typhimurium, Escherichia coli, Shigella flexneri, Proteus vulgaris, Serratia marcescens* | [26] |

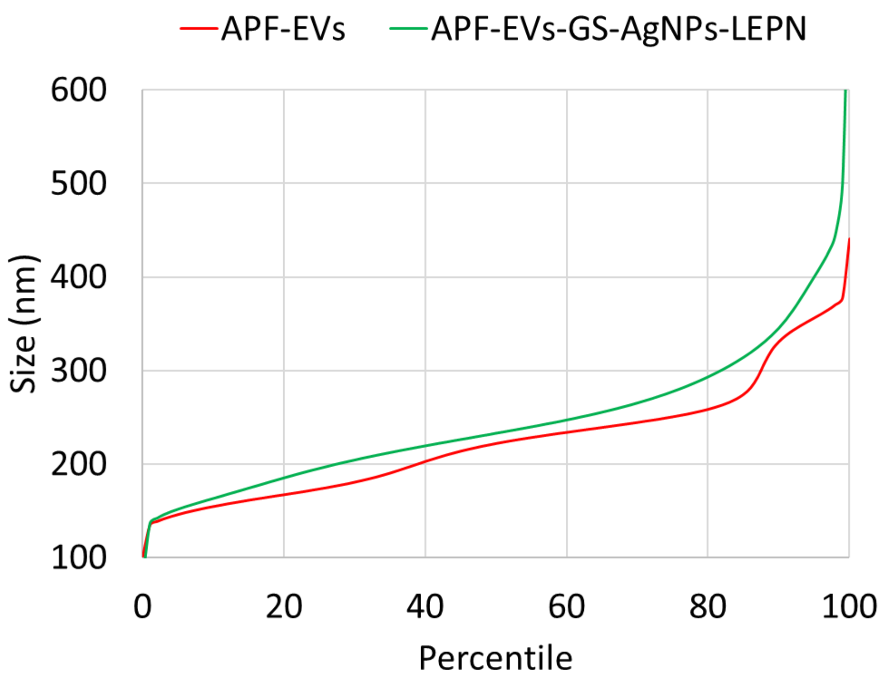


**Figure S1.** A representative graph showing the percentile of EVs in accordance with their size range. The majority of APF-EVs-GS-AgNPs-LEPN are shown have bigger size range as compared to the APF-EVs.

## References


1. Sgro, G.G.; Ficarra, F.A.; Dunger, G.; Scarpeci, T.E.; Valle, E.M.; Cortadi, A.; Orellano, E.G.; Gottig, N.; Ottado, J. Contribution of a harpin protein from Xanthomonas axonopodis pv. citri to pathogen virulence. Mol. Plant Pathol. **2012**, *13*, 1047–1059. https://doi.org/10.1111/j.1364-3703.2012.00814.x.
2. Dunger, G.; Garofalo, C.G.; Gottig, N.; Garavaglia, B.S.; Rosa, M.C.P.; Farah, C.S.; Orellano, E.G.; Ottado, J. Analysis of three Xanthomonas axonopodis pv. citri effector proteins in pathogenicity and their interactions with host plant proteins. Mol. Plant Pathol. **2012**, *13*, 865–876. https://doi.org/10.1111/j.1364-3703.2012.00797.x.
3. Guo, Y.; Figueiredo, F.; Jones, J.; Wang, N. HrpG and HrpX Play Global Roles in Coordinating Different Virulence Traits of Xanthomonas axonopodis pv. citri. Mol. Plant-Microbe Interact. **2011**, *24*, 649–661. https://doi.org/10.1094/MPMI-09-10-0209.
4. Dunger, G.; Arabolaza, A.L.; Gottig, N.; Orellano, E.G.; Ottado, J. Participation of Xanthomonas axonopodis pv. citri hrp cluster in citrus canker and nonhost plant responses. Plant Pathol. **2005**, *54*, 781–788. https://doi.org/10.1111/j.1365-3059.2005.01284.x.
5. Gottig, N.; Garavaglia, B.S.; Daurelio, L.D.; Valentine, A.; Gehring, C.; Orellano, E.G.; Ottado, J. Xanthomonas axonopodis pv. citri uses a plant natriuretic peptide-like protein to modify host homeostasis. Proc. Natl. Acad. Sci. USA **2008**, *105*, 18631–18636. https://doi.org/10.1073/pnas.0810107105.
6. Malamud, F.; Torres, P.S.; Roeschlin, R.; Rigano, L.A.; Enrique, R.; Bonomi, H.R.; Castagnaro, A.P.; Marano, M.R.; Vojnov, A.A. The Xanthomonas axonopodis pv. citri flagellum is required for mature biofilm and canker development. Microbiology **2011**, *157*, 819–829. https://doi.org/10.1099/mic.0.044255-0.
7. Baptista, J.C.; Machado, M.A.; Homem, R.A.; Torres, P.S.; Vojnov, A.A.; Amaral, A.M. do Mutation in the xpsD gene of Xanthomonas axonopodis pv. citri affects cellulose degradation and virulence. Genet. Mol. Biol. **2009**, *33*, 146–153. https://doi.org/10.1590/S1415-47572009005000110.
8. Tondo, M.L.; Petrocelli, S.; Ottado, J.; Orellano, E.G. The Monofunctional Catalase KatE of Xanthomonas axonopodis pv. citri Is Required for Full Virulence in Citrus Plants. *PLoS One* **2010**, *5*, e10803, doi:10.1371/journal.pone.0010803.

9. Kraiselburd, I.; Alet, A.I.; Tondo, M.L.; Petrocelli, S.; Daurelio, L.D.; Monzón, J.; Ruiz, O.A.; Losi, A.; Orellano, E.G. A LOV Protein Modulates the Physiological Attributes of Xanthomonas axonopodis pv. citri Relevant for Host Plant Colonization. PLoS ONE **2012**, *7*, e38226. https://doi.org/10.1371/journal.pone.0038226.
10. Strayer-Scherer, A.; Liao, Y.Y.; Young, M.; Ritchie, L.; Vallad, G.E.; Santra, S.; Freeman, J.H.; Clark, D.; Jones, J.B.; Paret, M.L. Advanced Copper Composites Against Copper-Tolerant Xanthomonas perforans and Tomato Bacterial Spot. Phytopathology® **2018**, *108*, 196–205, https://doi.org/10.1094/phyto-06-17-0221-r.
11. Paret, M.L.; Vallad, G.E.; Averett, D.R.; Jones, J.B.; Olson, S.M. Photocatalysis: Effect of Light-Activated Nanoscale Formulations of TiO2 on Xanthomonas perforans and Control of Bacterial Spot of Tomato. Phytopathology® **2013**, *103*, 228–236, https://doi.org/10.1094/phyto-08-12-0183-r.
12. Ocsoy, I.; Paret, M.L.; Ocsoy, M.A.; Kunwar, S.; Chen, T.; You, M.; Tan, W. Nanotechnology in Plant Disease Management: DNA-Directed Silver Nanoparticles on Graphene Oxide as an Antibacterial against Xanthomonas perforans. ACS Nano **2013**, *7*, 8972–8980, https://doi.org/10.1021/nn4034794.
13. Liao, Y.-Y.; Strayer-Scherer, A.L.; White, J.; Mukherjee, A.; De La Torre-Roche, R.; Ritchie, L.; Colee, J.; Vallad, G.E.; Freeman, J.H.; Jones, J.B.; et al. Nano-Magnesium Oxide: A Novel Bactericide Against Copper-Tolerant Xanthomonas perforans Causing Tomato Bacterial Spot. Phytopathology® **2019**, *109*, 52–62, https://doi.org/10.1094/phyto-05-18-0152-r.
14. Graham, J.H.; Johnson, E.G.; Myers, M.E.; Young, M.; Rajasekaran, P.; Das, S.; Santra, S. Potential of Nano-Formulated Zinc Oxide for Control of Citrus Canker on Grapefruit Trees. Plant Dis. **2016**, *100*, 2442–2447, https://doi.org/10.1094/pdis-05-16-0598-re.
15. Young, M.; Ozcan, A.; Myers, M.E.; Johnson, E.G.; Graham, J.H.; Santra, S. Multimodal Generally Recognized as Safe ZnO/Nanocopper Composite: A Novel Antimicrobial Material for the Management of Citrus Phytopathogens. J. Agric. Food Chem. **2017**, *66*, 6604–6608, https://doi.org/10.1021/acs.jafc.7b02526.
16. Adaskaveg, J.E. Copper Tolerance and Zinc Sensitivity of Mexican Strains ofXanthomonas campestrispv.vesicatoria,Causal Agent of Bacterial Spot of Pepper. Plant Dis. 1985, 69, https://doi.org/10.1094/pd-69-993.
17. Le Anh Dao, N.; Phu, T.M.; Douny, C.; Quetin-Leclercq, J.; Hue, B.T.B.; Bach, L.T.; Quynh Nhu, T.; Thi Bich Hang, B.; Thi Thanh Huong, D.; Thanh Phuong, N.; et al. Screening and comparative study of in vitro antioxidant and antimicrobial activities of ethanolic extracts of selected Vietnamese plants. Int. J. Food Prop. **2020**, *23*, 481–496, https://doi.org/10.1080/10942912.2020.1737541.
18. Braga Ribeiro, A.M.; de Sousa, J.N.; Costa, L.M.; Oliveira, F.A. de A.; dos Santos, R.C.; Silva Nunes, A.S.; da Silva, W.O.; Marques Cordeiro, P.J.; de Sousa Lima Neto, J.; de Siqueira-Júnior, J.P.; et al. Antimicrobial activity of Phyllanthus amarus Schumach. & Thonn and inhibition of the NorA efflux pump of Staphylococcus aureus by Phyllanthin. Microb. Pathog. **2019**, *130*, 242–246. https://doi.org/10.1016/j.micpath.2019.03.012.
19. Mazumder, A.; Mahato, A.; Mazumder, R. Antimicrobial potentiality of Phyllanthus amarus against drug resistant pathogens. Nat. Prod. Res. **2006**, *20*, 323–326. https://doi.org/10.1080/14786410600650404.
20. Sunitha, J. Antimicrobial Effect of Leaves of Phyllanthus niruri and Solanum nigrum on Caries Causing Bacteria: An In vitro Study. J. Clin. Diagn. Res. 2017, 11. https://doi.org/10.7860/JCDR/2017/23602.10066.
21. Ibrahim, D.; Hong, L.S.; Kuppan, N. Antimicrobial activity of crude methanolic extract from Phyllanthus niruri. Nat. Prod. Commun. **2013**, *8*, 493–496.
22. 22. Oyekanmi, B.A.; Osho, I.B. Antimicrobial, phytochemical and pharmacological properties of phyllanthus niruri Linn. FASEB J. **2016**, *30*, 1192.6.
23. Obiagwu, I.; Okechalu, O.; Njoku, M. Studies on Antibacterial Effect of the Leaves ff Phyllanthus Niruri on Some Enteric Pathogens. Niger. J. Biotechnol. **2011**, *23*, 22–27.

24. Shilpa, V.; Muddukrishnaiah, K.; Thavamani, Bs.; Dhanapal, V.; Arathi, K.; Vinod, K.; Sreeranjini, S. In vitro immunomodulatory, antifungal, and antibacterial screening of Phyllanthus niruri against to human pathogenic microorganisms. Environ. Dis. **2018**, *3*, 63. https://doi.org/10.4103/ed.ed_9_18.
25. Amin, Z.A.; Abdulla, M.A.; Ali, H.M.; Alshawsh, M.A.; Qadir, S.W. Assessment of In vitro antioxidant, antibacterial and immune activation potentials of aqueous and ethanol extracts of Phyllanthus niruri. J. Sci. Food Agric. **2012**, *92*, 1874–1877. https://doi.org/10.1002/jsfa.5554.
26. Natarajan, D.; Srinivasan, R.; Shivakumar, M.S. Phyllanthus wightianus Müll. Arg.: A Potential Source for Natural Antimicrobial Agents. BioMed Res. Int. **2014**, *2014*, 135082. https://doi.org/10.1155/2014/135082.
